# 非小细胞肺癌神经内分泌分化与其生物学特性及预后关系的研究

**DOI:** 10.3779/j.issn.1009-3419.2010.09.07

**Published:** 2010-09-20

**Authors:** 军 张, 锴 郑, 燕 郭, 鹏 张, 忠利 战

**Affiliations:** 1 300052 天津，天津医科大学总医院心胸外科 Department of Cardiothoracic Surgery, Tianjin Medical University General Hospital, Tianjin 300052, China; 2 300060 天津，天津医科大学肿瘤医院病理科 Department of Pathology, Cancer Institute and Hospital of Tianjin Medical University, Tianjin 300060, China

**Keywords:** 肺肿瘤, 神经内分泌分化, 生物学特性, 预后, Lung neoplasms, Neuroendocrine differentiation, Biological characteristics, Prognosis

## Abstract

**背景与目的:**

近年来研究发现非小细胞肺癌(non-small cell lung cancer, NSCLC)具有神经内分泌(neuroendocrine, NE)分化的特性。本研究旨在探讨NSCLC的神经内分泌分化情况及其生物学行为和预后的相关性。

**方法:**

随机收集2005年1月-2007年12月、随访资料满3年的206例NSCLC术后患者的病例及石蜡标本，所有患者术前均未经放化疗。石蜡标本采用EnVision免疫组化二步法检测NSCLC神经内分泌标记物神经元特异性烯醇化酶(neuronspecific enolase, NSE)、嗜铬素A(chromogranin A, CgA)及突触素(synaptophysin, Syn)的表达。所有数据均采用SPSS统计软件进行处理，同时采用*Kaplan*-*Meier*曲线描述生存率，并行*Log*-*rank*检验。

**结果:**

所有NSCLC病例中，82例伴有神经内分泌分化，阳性率为39.8%。CgA、NSE及Syn的阳性率分别为53例(25.7%)、104例(50.5%)和91例(44.2%)。统计学分析表明，非小细胞肺癌伴NE分化与癌细胞的分化程度相关，且Syn的阳性表达与癌细胞的分化程度及淋巴结转移情况相关。单因素生存分析结果经*Log*-*rank*检验示：Syn的阳性表达与患者术后生存率有关，而NE分化与患者术后生存率无明显相关性。

**结论:**

NSCLC是否伴有NE分化是判断其生物学行为的重要指标，而Syn可作为NSCLC伴NE分化的诊断标志物，Syn阳性表达提示患者预后不良。

## Abstract (en)

**Background and objective:**

Recently it has been proven that non-small cell lung cancer (NSCLC) also had the feature of neuroendocrine (NE) differentiation. The aim of this study is to investigate the correlation between NE differentiation of NSCLC and its biological behaviors, together with prognosis.

**Methods:**

All NSCLC paraffin-embedded specimens and cases, followed up over than 3 years, were randomly obtained from 206 patients from January 2005 to December 2007, who underwent surgical resection and confirmed pathologically. All of them were not underwent radiation and chemotherapy before operation. Immunohistochemical Envision two-step method was used to detect the expressions of NSE, CgA and Syn. And all data were analyzed using SPSS statistics software and *Kaplan*-*Meier* survival curves were constructed, and *Log*-*rank* test was also conducted.

**Results:**

Of the 206 patients, 84 cases with NE differentiation (39.8%) and CgA, NSE and Syn positive rates were 53 (25.7%), 104 (50.5%), 91 (44.2%) respectively; a statistically significant difference between NSCLC with NE differentitation were showed. The positive expression of Syn was closely correlated with histological differentiation, lymph node metastasis. The survival of single-factor analysis by the *Log*-*rank* test showed that Syn had relation to the postoperative survival rate of patients (χ^2^=4.164, *P*=0.041), while the relevance between patients with NE and survival had no significant difference (*P* > 0.05).

**Conclusion:**

NE differentiation is an important indicator of biological behavior of NSCLC; and the detection of Syn markers of neuroendocrine differentiation may be recommended to detect NE differentiation of NSCLC, and the positive expression of Syn suggests poor prognosis.

肺癌现已成为临床常见的恶性肿瘤之一，根据其细胞形态学、生物学和临床特征的不同可分为小细胞癌(small cell lung cancer, SCLC)和非小细胞肺癌(non-small cell lung cancer, NSCLC)。SCLC具有神经内分泌(neuroendocrine, NE)分化的特征，属于神经内分泌肿瘤。但近年来研究发现NSCLC同样也具有NE分化的特性^[1]^，并且WHO在1999年重新修订的肺与胸膜肿瘤组织病理学分类中增加了NSCLC伴神经内分泌分化的概念^[2]^，由于NSCLC占肺癌中绝大多数，故对其生物学特性及预后等的研究具有重要临床价值。本研究选取神经元特异性烯醇化酶(neuron-specific enolase, NSE)、突触素(synaptophysin, Syn)及嗜铬素A(chromogranin A, CgA)三种标记物联合应用，判断是否具有NE分化，并进一步探讨NE分化及这三种标记物的表达同NSCLC的生物特性及预后的相关性，从而为临床治疗及预后的评估提供依据。

## 1 材料与方法

### 1.1 临床资料

本研究的资料来源于天津医科大学附属肿瘤医院2005年1月-2007年12月、经外科手术治疗并病理证实为NSCLC，随访满3年的206例患者，所有患者术前均未经过放化疗。其中，男性112例，女性94例。年龄38岁-75岁，中位年龄61岁，临床分期为Ⅰ期-Ⅲa期，依据2004年版WHO最新肺癌病理学分类标准分类，其中鳞癌98例，腺癌76例，腺鳞癌20例，其它类型12例。所有标本均经10%福尔马林固定，常规石蜡包埋。

### 1.2 试剂和方法

#### 1.2.1 主要试剂

即用型鼠抗人NSE、CgA和Syn单克隆抗体的EnVision二步法试剂盒均由丹麦DAKO公司提供，购于基因科技(上海)有限公司。

#### 1.2.2 免疫组化EnVision二步法

石蜡连续切片5张，厚4 μm。1张用于HE染色，余供免疫组化用。石蜡切片预处理。二甲苯，乙醇梯度水化，染色前用1 mmol/L Tris-EDTA(pH9.0)进行高压加热修复抗原。切片0.3%H_2_O_2_避光处理20 min；然后PBS洗3次，每次5 min，加入一抗，将切片放入4 ℃冰箱过夜。转天取出湿盒，复温30 min后，PBS洗3次，每次5 min，加入二抗。室温放置30 min后，PBS洗3次，每次5 min。DAB显色5 min-10 min，蒸馏水冲洗。苏木素复染，脱水，封片。用已知阳性的类癌切片为阳性对照，并以切片中出现神经阳性为内对照，已知阳性切片以PBS代替一抗进行空白对照。

#### 1.2.3 阳性结果判断

背景清晰，胞浆内可见明确的棕黄色或棕褐色颗粒为阳性细胞，每例随机计数10个高倍视野(×400)，阳性染色的癌细胞≤5%视为阴性(-)＞5%视为阳性(+)，其中阳性癌细胞5%-10%为(+)，11%-20%(++)，21%-50%(+++)。有下述情况之一者确定伴NE分化：有一种NE标记物—表达为(+++)，或至少有两种标记物表达为(+-++)。

### 1.3 随访

所有患者以病例跟踪，电话随访为主，信件随访辅助。随访时间从手术之日开始至死亡或是最后1次随访，以月为单位计算，随访满3年截止。

### 1.4 统计方法

所有数据均采用SPSS 13.0统计软件进行分析。应用R×C列联表χ^2^检验，比较三种NE标记物及NE与临床生物学特征的关系。单因素生存分析采用*Kaplan*-*Meier*曲线描述生存率，并行*Log*-*rank*检验。*P*＜0.05表示差异有统计学意义。

## 2 结果

### 2.1 NE标记物在NSCLC中的表达

三种神经内分泌标记物的阳性部位均为胞浆着色，呈棕色颗粒。NSE、Syn多呈弥漫性胞浆着色(图 1)，CgA呈灶性分布或为弥漫性着色(图 2)。依据至少2种NE标记物阳性的病例诊为伴有NE分化的NSCLC的判断标准，206例NSCLC中伴神经内分泌分化者82例(占39.8%)，不伴神经内分泌分化者124例；CgA阳性例数为53例(25.7%)，NSE阳性例数为104例(50.5%)，Syn阳性例数为91例(44.2%)。

**1:**
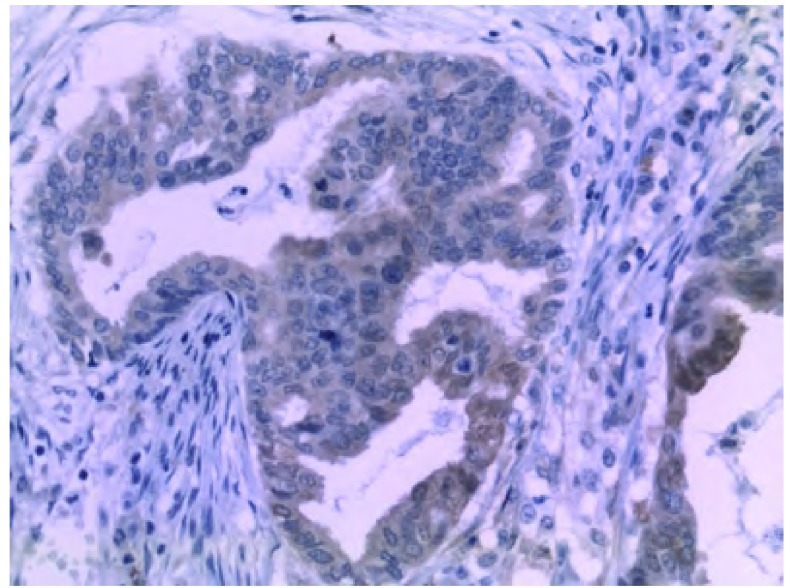
Syn在肺腺癌中的表达(EnVision, ×400)。Syn：突触素。 Syn positive staining in lung adenocarcinoma (EnVision, ×400). Syn: synaptophysin.

**2:**
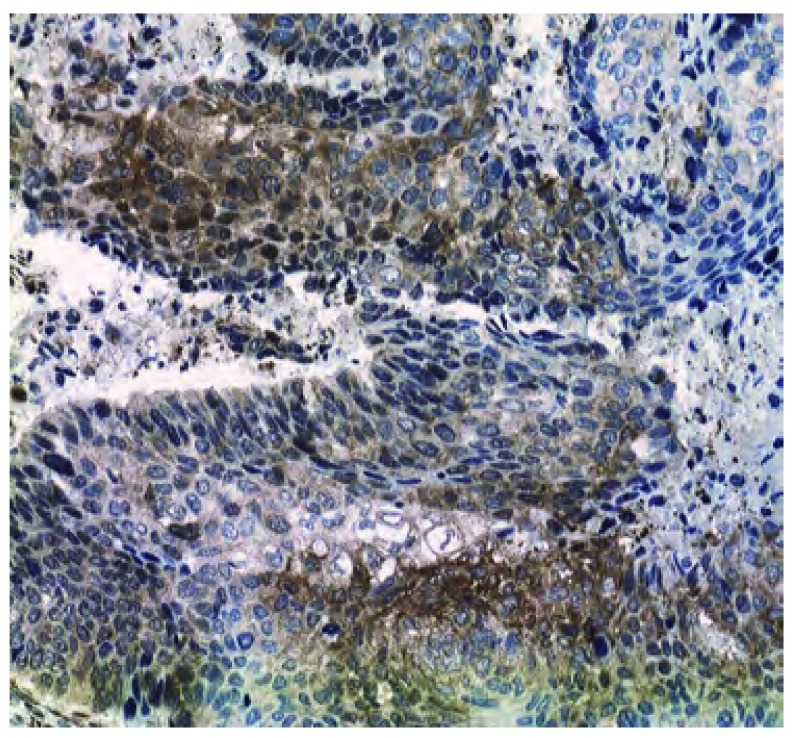
CgA在鳞癌中的表达(EnVision，×200)。CgA：嗜铬素A。 CgA positive staining in squamous cell carcinoma (EnVision, ×200). CgA: chromogranin A.

### 2.2 组织分型与NE分化及其标记物的表达

不同组织分型的肺癌中NE分化及其标记物表达的情况见表 1，经统计学分析表明，NE分化与临床病理类型无相关性。

**1:** 各型NSCLC的Syn、CgA和NSE的阳性率 The positive rates of CgA、NSE and Syn

Type	The positive rate of NE marker (%)			NE differentiation
	CgA	NSE	Syn	
Squamous cell carcinoma	20 (24.4)	40 (48.8)	36(17.4)	33 (40.2)
Adenocarcinoma	24 (28.2)	46 (54.4)	45 (52.9)^*^	36 (42.3)
Adenosquamous carcinoma	4(18.2)	10 (45.5)	5 (22.7)	7(31.8)
Others	5 (29.4)	8(47.1)	5 (29.4)	6(35.3)
^*^The positive rate of Syn expression among all subtypes of NSCLC has statistical difference, *P*=0.041.				

### 2.3 淋巴结转移与NE分化及其标志物的表达

三组标记物中，Syn阳性表达在淋巴结转移组显著高于无淋巴结转移组(*P*=0.028)；有无淋巴结转移与NE分化间无相关性(表 2)。

**2:** 淋巴结转移情况与NE分化相关性 The relationship between Lymph node metastasis and NE differentiation

Metastasis	The positive rate of NE markers (%)			NE differentiation
	CgA	NSE	Syn	
Yes	24 (26.4)	46 (50.5)	48(52.7)^*^	40 (44.0)
No	29(25.2)	58 (50.4)	43 (37.4)	42 (36.5)
Total	53 (25.7)	104(50.5)	91 (44.2)	82 (39.8)
^*^The positive rate of Syn expression in the two groups has statistical difference, *P*=0.028.				

### 2.4 肿瘤分化程度与NE标记物的表达

NE分化的阳性率和其标记物在低分化的NSCLC中表达较高，中分化组明显升高；Syn的阳性率表达在三组间的差异有统计学意义(*P*＜0.05)(表 3)。

**3:** NE标记物阳性率与NSCLC分化程度的关系 The relationship between positive rate of NE and histological differentiation

Differentiation degree	The positive rate of NE markers (%)			NE differentiation
	CgA	NSE	Syn	
High	15 (24.2)	32(51.6)	23 (40.3)	18 (29.0)
Moderate	21 (27.6)	37 (48.7)	29 (42.1)	29 (38.2)
Low	17(25.0)	35 (51.5)	39 (50.0)	35 (51.5)
*χ*^2^	0.239	0.157	7.164	6.951
*P*	0.887	0.540	0.028^*^	0.031^*^
^*^P＜0.05.				

### 2.5 生存分析

所有NSCLC患者随访结果显示，伴有NE分化82例，其中死亡35例，3年生存率为57.3%；不伴有NE分化124例，其中死亡34例，3年生存率为68.5%。经*Log*-*rank*检验差异无统计学意义(*P*＞0.05)。

CgA和NSE的阳性表达与患者预后亦无相关性(*P*＞0.05)；但Syn的阳性率与患者的生存期之间的差异具有统计学意义(χ^2^=4.164, *P*=0.041)(图 3)。

**3:**
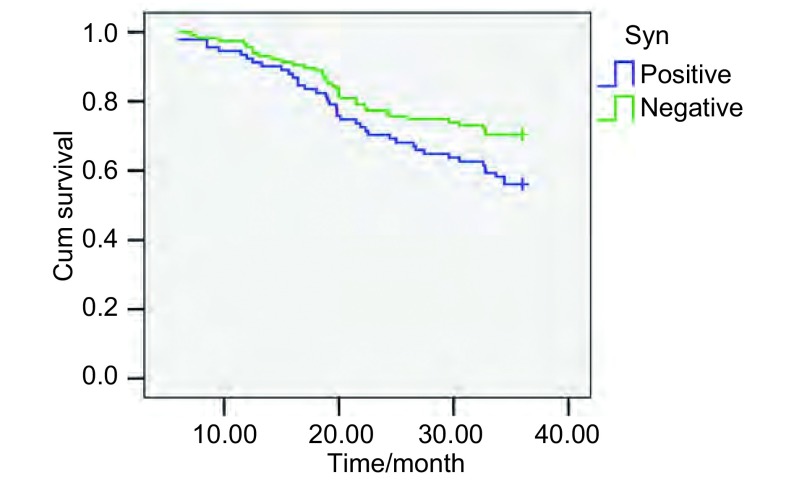
Syn分化与患者的生存曲线 Surivival curves of patients with Syn of non-small cell lung cancer

## 3 讨论

大量研究已经证实，人体肺脏是一个内分泌器官，SCLC属神经内分泌癌。但近年来发现NSCLC中也存在着不同程度的NE标志^[3-5]^。1999年重新修订的“肺与胸膜肿瘤组织病理学分类标准”中新增加了NSCLC伴NE分化的概念。并指出其特异性标记物为CgA与Syn。2003年神经内分泌癌/分化的判断指出：肿瘤组织中，一种NE标记物明确阳性，且阳性细胞数＞50%时可诊断为神经内分泌癌，否则为伴NE分化。

目前，研究NE的方法有多种^[6]^，如：生物素检测系统、电镜/免疫组织化学法等，其中Slodkowsk等^[7]^研究认为免疫组化为检测NE分化的标准方法。本研究采用EnVision检测系统，原理是EnVision复合物与已结合抗原的标的一抗结合，形成含酶(HRP或AP)的抗原抗体多聚复合物。该系统较传统的三步法敏感性增强，由于不再是生物素标记二抗与链亲和素标记的三抗结合，所以整个系统不受内源性生物素的干扰，减低了背景。

尽管有众多NE分化标志抗原和免疫组化技术的不断发展，但由于抗原含量甚微或是其前体分子较大，使针对这些抗原的抗体敏感性受限；同时抗原存在交叉反应，使抗体的特异性不够理想^[8]^。目前认为NSE、CgA及Syn的组合是一组特异性和敏感性较好的NE分化的共同标志，可作为标准的检测指标。本研究结果为NSCLC伴NE分化阳性率为39.8%，NSE阳性率50.5%，Syn阳性率44.2%，CgA阳性率25.7%，这与相关文献的研究结果相近。同时，Syn阳性率随肿瘤细胞分化程度降低而升高，差异具有统计学意义。Syn阳性表达率与NE阳性表达率相近，所以Syn可作为诊断NSCLC伴NE分化的主要标志物。

同时CgA、NSE及Syn三种标记物的敏感性及特异性有所不同。NSE阳性表达例数多于后两者，但其在不同组别间的阳性差异却较后两者小，说明其虽然敏感性高，但特异性较差。相比之下，CgA和Syn的特异性较高，但CgA的敏感性较Syn差，CgA阳性表达的53例中有50例Syn亦为阳性，提示凡CgA能够识别的NE分化者，Syn几乎也能够判定；故而我们认为联合应用NSE及Syn是判断是否具有NE分化的较为可靠的指标。

本研究结果发现，NE分化与术后淋巴结转移、肿瘤分化程度相关。NE分化与术后淋巴结转移情况无相关性，但淋巴结转移组的Syn阳性率显著高于无淋巴结转移组(*P*＜0.05)。NSCLC的分化程度越低，伴NE分化的阳性率越高。故可认为肿瘤的分化程度对NE的诊断和Syn的阳性率均有着非常重要的意义。所以NSCLC伴NE分化与肿瘤的分化程度相关。但我们认为NE分化同生存期并无相关性，而Syn阳性表达作单因素生存分析发现，Syn标记物的阳性表达与生存期相关(*P*=0.041)，随着患者生存时间的延长，这种差异越明显。这与Howe等^[9]^的研究结果相符。这一结果提示Syn对NSCLC患者的临床和预后的评估有起着关键的作用。Gonzalez等^[10]^研究Ⅰ期鳞癌及腺癌患者中，结果Syn阳性表达患者的肿瘤更具侵袭性，预后更差。本研究中，NSCLC低分化组的Syn阳性率表达均高(*P*＜0.05)。说明随着Syn阳性率的升高，肿瘤的侵袭性增强。

综上所述，NSCLC伴NE分化与其肿瘤细胞的分化程度相关，肿瘤细胞分化越低，NE阳性表达率越高。肿瘤低分化患者Syn阳性表达率与NE阳性表达率接近(两种标记物同时表达为阳性)。故Syn可作为NSCLC伴NE的诊断标志物。同时，Syn标记物的阳性表达与生存期相关，可为临床对患者的诊治及预后评估提供依据。在NSCLC中NE分化同肿瘤的多种生物学行为相关，可以作为判定肿瘤转移潜能的独立指标。
